# Etiological Diagnosis and Personalized Therapy for Hypertension: A Hypothesis of the REASOH Classification

**DOI:** 10.3390/jpm13020261

**Published:** 2023-01-30

**Authors:** Chong Xu, Moran Li, Weilun Meng, Jun Han, Song Zhao, Jiamin Tang, Haotian Yang, Rusitanmujiang Maimaitiaili, Jiadela Teliewubai, Shikai Yu, Chen Chi, Ximin Fan, Jing Xiong, Yifan Zhao, Yawei Xu, Yi Zhang

**Affiliations:** Department of Cardiology, Shanghai Tenth People’s Hospital, Tongji University School of Medicine, Shanghai 200072, China

**Keywords:** etiological diagnosis, personalized therapy, hypertension, REASOH classification

## Abstract

With the epidemic of risk factors such as unhealthy lifestyle, obesity and mental stress, the prevalence of hypertension continues to rise across the world. Although standardized treatment protocols simplify the selection of antihypertensive drugs and ensure therapeutic efficacy, the pathophysiological state of some patients remains, which may also lead to the development of other cardiovascular diseases. Thus, there is an urgent need to consider the pathogenesis and selection of antihypertensive drug for different type of hypertensive patients in the era of precision medicine. We proposed the REASOH classification, based on the etiology of hypertension, including renin-dependent hypertension, elderly-arteriosclerosis-based hypertension, sympathetic-active hypertension, secondary hypertension, salt-sensitive hypertension and hyperhomocysteinemia hypertension. The aim of this paper is to propose a hypothesis and provide a brief reference for the personalized treatment of hypertensive patients.

## 1. Introduction

Hypertension, the most common chronic non-communicable disease worldwide, remains the most prevalent risk factor for cardiovascular and cerebrovascular death. According to the World Health Organization (WHO) [1], an estimated 1.28 billion adults worldwide have arterial hypertension, but only half have been properly diagnosed and controlled. With the widespread prevalence of risk factors such as unhealthy lifestyle, smoking, and obesity, the estimated prevalence and control of hypertension is still not optimistic, causing the grim situation of blood-pressure control [1].

The evidence-based international strategies for the control and treatment of hypertension focus on using standardized treatment protocols to achieve the blood-pressure (BP) target range. Hypertension is traditionally divided into essential and secondary hypertension. Patients with primary hypertension account for more than 90% of all hypertensive patients, and the etiology is mostly unclear. Therefore, the application of standardized treatment protocols can significantly simplify the selection of anti-hypertensive drugs and ensure therapeutic effectiveness. For example, the 2020 ISH hypertension guidelines recommend a four-stage, streamlined treatment protocol [2]. 

**Figure 1:**
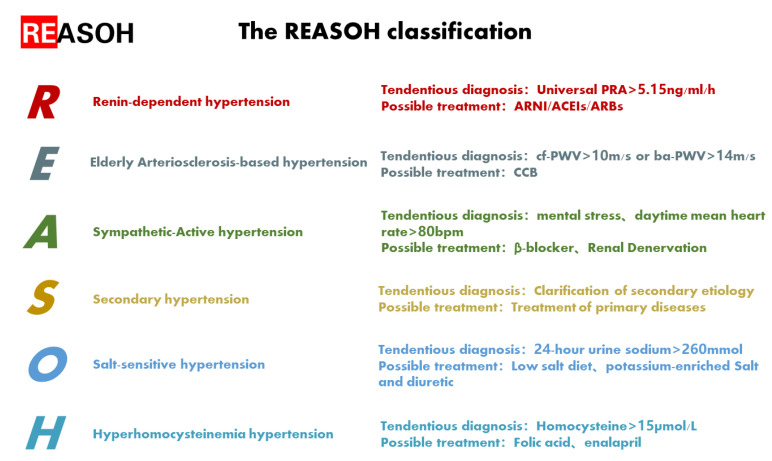
The REASOH classification: a hypothesis about etiological diagnosis and personalized therapy for hypertension. ACEIs: angiotensin-converting enzyme inhibitors; ARBs: angiotensin-receptor blockers; ARNI: angiotensin receptor blocker and neprilysin inhibitor; ba-PWV: brachial-ankle pulse wave velocity; CCB: calcium channel blocker; cf-PWV: carotid-femoral pulse wave velocity; PRA: plasma renin activity.

Nonetheless, although non-etiological treatments can control BP, the pathophysiological state of some patients remains, which may also lead to the development of heart failure, chronic kidney disease and other diseases [3,4]. Therefore, there is an urgent need to find a system of diagnosis and treatment based on etiology to improve the prognosis of hypertensive patients. However, since the current evidence is insufficient, we mainly propose a hypothesis named the REASOH classification. The REASOH classification (Figure 1) divides hypertensive etiology into six categories: renin-dependent hypertension, elderly arteriosclerosis-based hypertension, sympathetic-active hypertension, secondary hypertension, salt-sensitive hypertension, and hyperhomocysteinemia hypertension. The classification is primarily applicable to untreated hypertensive patients without a compelling indication for a specific treatment. Different diagnostic and therapeutic approaches are summarized for each type. Noteworthily, the pathogenesis of hypertension is multifactorial and the REASOH classification lacks sufficient clinical studies for support. Therefore, this hypothesis allows only a cursory assessment of the pathogenesis of hypertensive patients, which may provide a step forward for the hypertension treatment in the era of precision medicine. An overview of the REASOH classification in hypertensive patients is presented.

## 2. Renin-Dependent Hypertension

### 2.1. Definition and Pathogenesis

Renin-dependent hypertension is defined as high BP caused by elevated plasma renin activity (PRA). Hypertensive patients with high PRA are common, and it is highly related to the poor prognosis of cardiovascular diseases such as myocardial infarction [5,6,7,8]. It was demonstrated that activation of the renin-angiotensin-aldosterone system (RAAS) was strongly associated with elevated BP [9,10,11]. However, PRA was not high, and even decreased in some hypertensive patients, which might be related to excessive volume loading [12]. Therefore, renin-dependent hypertension may be identified by effectively measuring PRA in clinical practice [13].

### 2.2. Diagnosis and Hypertension-Mediated Organ Damage

At present, the diagnostic criteria for renin-dependent hypertension are controversial. Laragh et al. reported that a reliable indicator of renin-dependent hypertension was for PRA ≥ 0.65 ng/mL/h [13]. However, PRA is susceptible to the pharmacological effects of anti-hypertensive drugs. For example, diuretics can increase PRA by reducing systemic volume, while beta-blockers (BB) reduce PRA by decreasing sympathetic tension [14]. In addition, when patients are taking angiotensin-converting enzyme inhibitors (ACEIs) or angiotensin-receptor blockers (ARBs), the true PRA can be overestimated by approximately 10% of the measured value [15]. Therefore, it is necessary to estimate the effective PRA based on patients’ current oral medication. In terms of hypertension-mediated organ damage, Tomasz et al. found that increased PRA was associated with more severe arterial stiffness and impaired renal function [16].

### 2.3. Treatment

It is recommended that patients with suspected renin-dependent hypertension should take anti-RAAS drugs as a priority, including ACEIs, ARBs and angiotensin receptor-neprilysin inhibitors (ARNIs) [17,18]. Additionally, BB is used as a second-line treatment by inhibiting the central and peripheral RAAS, suppressing myocardial contractility and slowing down the heart rate [19]. Egan et al. found that a renin test-guided therapeutic algorithm could control BP equally well or better than clinical hypertension specialists’ care [20]. In contrast, in a small cohort of hypertensive patients, Leotta et al. found that the use of PRA did not optimize the rate of BP control [21]. Therefore, a larger sample size may be required to assess the clinical application of PRA. Meanwhile, in hypertensive patients with renin-dependent vasoconstriction, BP can be lowered most effectively by anti-renin or anti-angiotensin therapies. In contrast, hypertensive patients with low PRA were susceptible to an elevated BP response with anti-RAAS drugs [22]. Therefore, dynamic monitoring of PRA is essential to guide BP management in renin-dependent hypertensive patients. Moreover, the financial cost should also be taken into consideration in BP control. While the PRA-guided therapy increased the cost of care for hypertensive patients in the short term, it might be more cost-effective in the long run [22]. To some extent, the PRA-guided therapy provides more precise and individualized drug selection for hypertensive patients. 

## 3. Elderly Arteriosclerosis-Based Hypertension

### 3.1. Definition and Pathogenesis

According to the 2017 clinical practice guidelines for hypertension of the American College of Cardiology and the American Heart Association, elderly hypertension is defined as hypertension in people whose age ≥ 65 years with systolic BP ≥ 140 mmHg and/or diastolic BP ≥ 90 mmHg on three or more non-same-day measurements [23]. Compared with 32% of adults aged 40–59, there are 70% of older adults suffering from hypertension in the United States [24]. With the gradual aging of the global population, the prevalence of hypertension will continuously increase [25]. In terms of pathophysiological features, elastic arteries undergo vascular dilatation and stiffness with aging, which is manifested as vasomotor dysfunction. In addition, neurohormonal, autonomic dysregulation, and renal aging are also involved in the development of hypertension in the elderly [26,27].

### 3.2. Diagnosis and Hypertension-Mediated Organ Damage

The characteristics of elderly hypertension mainly include elevated systolic BP, increased pulse pressure (PP), high BP variability and postural hypotension. Among them, PP difference is often considered as a proxy for arterial stiffness in the elderly [28]. However, since PP difference is influenced by both cardiac and arterial function, a more accurate criterion for assessing arterial stiffness is arterial pulse wave velocity (PWV). Carotid-femoral pulse wave velocity (cf-PWV) > 10 m/s or brachial-ankle pulse wave velocity (ba-PWV) ≥ 18 m/s are defined as arterial stiffness [29,30]. PWV has been shown to be strongly associated with the progression of BP [31,32]. Additionally, elderly hypertension combined with cf-PWV > 10 m/s or ba-PWV ≥ 18 m/s may be diagnosed as the elderly arteriosclerosis-based hypertension. Furthermore, elderly people with hypertension are more likely to develop cardiovascular diseases such as myocardial infarction, heart failure and stroke [33]. In addition, cognitive decline, dementia, and decreased activity function are also common comorbidities of elderly hypertension, which together lead to a decrease in the quality of life [34,35].

### 3.3. Treatment

Given the characteristics of elderly hypertension, including high BP variability and multiple comorbidities, the ideal anti-hypertensive drug should have the advantages of stable and effective BP reduction, few adverse reactions and easy administration. Previous studies have shown that calcium channel blockers or diuretics can be used as initial drug therapy or long-term maintenance in the elderly [36,37]. Moreover, ACEIs/ARBs or BB can enhance the anti-hypertensive effect and reduce adverse reactions when combined with the above drugs.

## 4. Sympathetic-Active Hypertension

### 4.1. Definition and Pathogenesis

Sympathetic-active Hypertension (SAH), characterized by sympathetic activity (SA) activation and enhancement, has diverse clinical phenotypes, including marginal hypertension, elderly-systolic hypertension, white-coat hypertension, concealed hypertension, obstructive-sleep-apnea-related hypertension, psychological-pressure-related hypertension, the hypertensive state in youth induced by hyperkinesia, and so on [38]. Epidemiological data have shown that overactivation of the sympathetic nervous system (SNS) generally exists in patients with essential hypertension, accounting for 14–44% [38,39]. Currently, SAH is considered to be related to a series of pathophysiological mechanisms such as stress response, overactivation of RAAS, metabolic dysfunction, inflammatory immunity, dysfunction of arterial baroreceptors and chemoreceptors, and abnormal neural regulation [40]. In addition, SAH has a strong association with some cardiovascular risk factors such as high-salt diet, obesity and smoking [41].

### 4.2. Diagnosis

At present, for the screening of SAH, some clinical assessments for SA have been proved to be effective, including clinical features, neurotransmitter detection, metabolic biochemical-index detection, sympathetic-nerve-potential detection, and hemodynamic detection [42]. Studies have shown that patients who meet the following description are suggestive of the possibility of SAH, requiring further examination and early intervention: (1). Atypical hypertension: severe, unstable or refractory hypertension, paroxysmal hypertension, hypertension resistant to ACEI and diuretic combination, hypertension with sudden onset in youth, hypertension associated with sympathomimetic drugs, etc. (2). Certain hypertension complications: acute stroke, alcohol abuse, withdrawal syndrome, and sleep apnea syndrome, etc.

Sympathetic-nerve-potential detection is the gold standard for the evaluation of SA. However, the clinical application is limited by the invasion operation and high requirements for the equipment. In recent years, noninvasive electro-facial nerve imaging, a modified method of measuring sympathetic skin response to reflect the functional status of sympathetic postganglionic fibers, has shown clinical promise in the assessment of SA [43]. Furthermore, SA can be evaluated by measuring neurotransmitters directly or indirectly. Most studies have detected norepinephrine spillover in evaluating SA, but the proportion of NE released through synapses into the systemic circulation is restricted, so it can only indirectly reflect part of the sympathetic activation and abnormal enhancement state [44].

In addition, resting heart rate (rHR) and heart rate variability (HRV) can also indirectly reflect SA. rHR has a good correlation with plasma NE level, which can better reflect SA [45]. The 2018 ESC Guidelines for Hypertension state that rHR is a risk factor for cardiovascular disease in patients with hypertension, and that rHR > 80 beats/min is one of the risk predictors of adverse cardiovascular events. Grassi et al. have shown that rHR > 80 beats/min is significantly associated with an increase in left-ventricular-mass index, muscle sympathetic-flow and venous plasma-NE levels [46]. Meanwhile, the relationship between sympathetic activation and heart rate assessed by the micro-neuroimaging technique is more significant than that between plasma norepinephrine and heart rate [46]. These results indicate that hypertensive patients with HR > 80 beats/min are likely to have sympathetic overactivation. However, the above methods are controversial, and more studies are needed to verify them. Both ventricular hypertrophy and sympathetic activation are involved in the increased cardiovascular risk in patients with SAH.

### 4.3. Treatment

#### 4.3.1. Drug Therapy

Ideally, antihypertensive agents should be individualized based on the underlying mechanism of hypertension. BB can reduce heart rate and cardiac output, inhibit renin secretion, and have anti-anxiety effects. They can reduce BP by decreasing epinephrine secretion, especially for anxious patients with sinus tachycardia. Therefore, BB may be the primary option for patients with SAH. On the contrary, Lindholm et al. found that the effect of BB is less than optimum, with a raised risk of stroke compared to other antihypertensive drugs in a meta-analysis [47]. However, the study has some limitations. The results were mainly based on atenolol, and did not fully reflect the therapeutic effects of other highly selective BB. Moreover, the analysis included mainly elderly patients with hypertension, and could not be generalized to the whole population. Therefore, different populations and drugs are needed to validate the results. Given that sympathetic excitation often co-exists with RAAS and/or volume-mediated mechanisms causing essential hypertension, α/β blockers can be prescribed alongside drugs targeting RAAS and volumetric mechanisms in these patients [38].

#### 4.3.2. Non-Drug Therapy

Renal denervation (RDN) is an emerging technique to block the sympathetic efferent and afferent fibers by damaging the renal sympathetic fibers, which are distributed around the vasculature. In SAH patients, renal SA could be relieved by removing renal sympathetic innervation. RDN is indicated for hypertensive patients with significant sympathetic excitatory properties, such as 24 h average heart rate (without drug treatment) ≥ 80 beats/min, and resistant hypertension patients with the maximum tolerated dose of A+C+D therapy [48,49]. In addition, other therapies such as baroreflex-activation therapy have also been shown to be effective for SAH in clinical studies [50,51].

## 5. Secondary Hypertension

### 5.1. Definition and Pathogenesis

Secondary hypertension, accounting for approximately 10% of all hypertension, which is a subtype of hypertension with a clear and reversible underlying cause, is usually associated with a higher risk of cardiovascular events [2]. In clinical practice, early identification and intervention of secondary hypertension can help control blood pressure, reduce the use of hypertension drugs, and even reverse or cure secondary hypertension. The etiological types of secondary hypertension are varied. At present, in addition to the diet and medication causes, the most common causes include aortic coarctation, renal artery stenosis, renal parenchyma disease, primary hyperaldosteronism, pheochromocytoma, Cushing’s syndrome, aortic stenosis, obstructive sleep apnea, and thyroid disease [52]. In this part, we focus on the identification of common causes of secondary hypertension, and summarize the treatment recommendations on the basis of the etiological classification.

### 5.2. Screening and Diagnosis

Since only 10% of hypertensive patients may have secondary hypertension, widespread secondary hypertension screening should not be recommended for every patient. We recommend routine screening only for those with one or more of the following conditions: sudden onset of acute hypertension in patients with severe resistant hypertension or long-term stable blood pressure; young (age < 30) patients with hypertension, especially those without hypertension risk-factors (obesity, metabolic syndrome, family history, etc.); hypertension with target organ damage or sleep apnea; hypertension with acid-base imbalance and electrolyte disturbances, such as hypokalemia and metabolic alkalosis; hypertension with clinical features related to secondary etiology.

**Figure 2:**
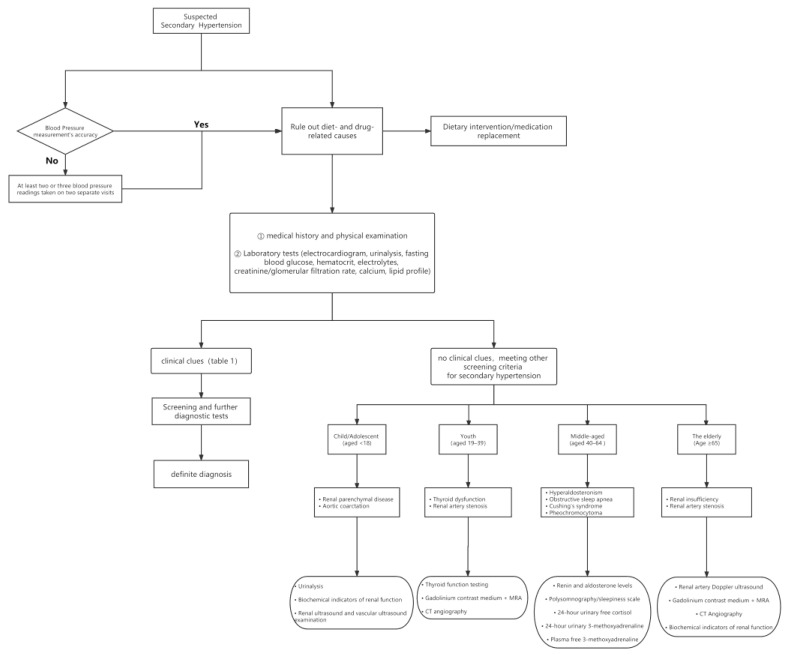
Recommended screening process for patients with suspected secondary hypertension. CT: computed tomography; MRA: magnetic resonance angiography.

The distribution of potential causes of secondary hypertension varies with age. Renal parenchymal disease and aortic coarctation are the most common causes of hypertension in adolescents younger than 18 years of age. For young patients aged 19–39, it is required for them to be screened for thyroid function. For patients aged 40–64, it is necessary to screen for primary aldosteronism (PA), thyroid dysfunction, obstructive sleep apnea hypopnea syndrome (OSAHS), Cushing’s syndrome, and pheochromocytoma. For patients aged over 65, renovascular hypertension, PA, OSAHS and renal-parenchyma hypertension are common subtypes of secondary hypertension [53]. Figure 2 provides a common screening procedure for patients with suspected secondary hypertension.

### 5.3. Treatment

In addition to early identification and diagnosis of secondary hypertension, the selection of appropriate treatment according to the etiology and subtypes can help the patients obtain the maximum benefits. ACEIs or ARBs is preferred as the first-line medication for renal parenchyma hypertension. For renovascular hypertension, antihypertensive, cholesterol-lowering and antiplatelet drugs are the foundation, and percutaneous stent implantation is the first choice for emergency interventional treatment, which is applicable to atherosclerotic renovascular stenosis but not to fibromuscular dysplasia. Sleep-apnea-associated hypertension can be treated with non-invasive positive pressure ventilation. Surgical resection is the first choice for PA, pheochromocytoma, and Cushing’s syndrome. Moreover, endovascular intervention is recommended for aortic coarctation [2,54,55,56,57]. The details are shown in Table 1.

## 6. Salt-Sensitive Hypertension

### 6.1. Definition and Pathogenesis

Based on a scientific statement from the American Heart Association, salt-sensitive hypertension (SSH) is defined as a marked increase in blood pressure in response to increased salt intake. In contrast, salt-resistant hypertension (SRH) does not present in this way [58]. Salt sensitivity related to BP appears to have become a major public-health problem, with an estimated incidence of 30–50% in the hypertensive population and 25% in the normotensive population [59]. The relationship between salt intake and blood pressure and its specific mechanisms have been investigated in numerous experimental and clinical studies [60]. For example, the Intersalt Cooperative Research Group found that individual 24-hour urinary sodium excretion higher than 100 mmol was related to elevated systolic/diastolic BP on average by 3/0 to 6/3 mmHg [61]. Mechanistically, some evidence strongly demonstrates that genetic mechanisms partly determine the effect of salt intake on BP, such as the nitric oxide synthase 3 (*NOS3*) gene, angiotensinogen (*AGT*) gene, G protein subunit beta 3 (*GNB3*) gene and so on [62]. In addition, the high sodium diet is involved in the development of SSH by activating the RAAS and sympathetic nervous system, mediating insulin resistance and peripheral vascular-pressure resistance [63,64,65,66]. 

### 6.2. Diagnosis and Hypertension-Mediated Organ Damage

There are no optimal criteria for the diagnosis of SSH currently. However, the following two methods are commonly used. The first approach is the chronic-salt-load test, which measures changes in BP for subjects on alternating low-salt and high-salt diets. The Grim and Weinberger’s protocol is commonly used. Specifically, participants were required to follow 5 days of a high-salt diet (≥200 mmol/day) and then 7 days of a low-salt diet (≤15 mmol/day). BP and urinary sodium were recorded daily. Salt sensitivity (SS) was defined as a decrease in mean arterial-pressure equal to or greater than 10 mmHg at the end of the low-salt diet [67]. The second approach is the acute-salt-load test. Participants were given saline for sodium load followed by a low-sodium diet combined with diuretic treatment for sodium and volume depletion. Mean arterial pressure was measured for both phases, to identify SSH [59]. Nevertheless, these methods are not widely used in clinical practice, due to the complicated procedures and limited compliance of the subjects. Furthermore, Castiglioni et al. proposed the classification of SS risk-level based on the 24-hour ambulatory BP and heart rate. The result was consistent with the traditional method of diagnosis, but further validation is required by additional prospective clinical studies [68]. Due to the high price and difficult implementation of diagnostic methods for SSH, the combination of high urinary-sodium with low plasma-renin may be a more appropriate diagnostic clue for salt-sensitive hypertension. The 24-hour urine-sodium (UNa) test is a common method for assessing salt-intake levels. Notably, there is variability in urinary-sodium excretion, and many hormones such as aldosterone, cortisol or cortisone are involved in the process, in addition to sodium intake [69]. Given the inconvenience of collecting 24-hour urine from outpatients, the main method commonly used to assess 24-hour UNa is the spot-urine method. The Kawasaki method, Tanaka method and Intersalt method are usually used in Asian populations. Among them, the Kawasaki method is the least biased in the Chinese population [70]. Therefore, the Kawasaki method may be used for assessing salt-intake levels to screen for SSH in hypertensive patients. Of concern is the fact that, 24-hour urine collection or spot-urine methods may overestimate or underestimate dietary-sodium intake, so a combined assessment of multiple measures is necessary.

SSH is an independent risk factor for cardiovascular events in several cohort studies [71,72]. A high-salt diet can impair vascular endothelial function by reducing nitric oxide bioavailability and mediating oxidative stress, resulting in vascular stiffness [73,74]. The latest meta-analysis showed that for every 89.3 mmol/day of salt-intake reduction by individuals, PWV would decrease by 2.84% [75]. Elevated levels of dietary Na^+^ can suppress RAAS and activate epithelial Na^+^ channels to reduce Na+ reabsorption, which ultimately lead to kidney damage [73]. Additionally, urinary albumin excretion is higher in SSH patients compared to patients with SRH [76]. In a randomized double-blind crossover trial, it was found that restricting salt intake can reduce urinary albumin excretion and improve glomerular filtration rate (GFR) in hypertensive patients [77].

### 6.3. Treatment

To date, there are no definitive therapeutic criteria for patients with SSH. However, it is essential to control salt intake. The average salt intake of the global population is roughly 9-12 g/day, while the World Health Organization limits salt intake to no more than 5 g/day [78]. The guidelines for the management of hypertension in China stated that the salt intake of hypertensive patients should not exceed 6 g/day [79]. In addition, weight control (body mass index < 25 kg/m^2^), increasing potassium intake, limiting alcohol intake and doing regular exercise can also control blood pressure [80]. In clinical practice, there are also discussions about which medications to choose for SSH patients. The latest meta-analysis compared the effects of different medications and dietary regimens on BP in SSH patients, and found that CCB combined with thiazide diuretics and moderate salt-intake (mean salt-intake of 7.02 g/day) was most effective in controlling BP [81]. However, the first-line pharmacological treatment of SSH is still controversial, and needs to be verified in more clinical studies.

## 7. Hyperhomocysteinemia Hypertension

### 7.1. Definition and Pathogenesis

According to the 2016 Chinese evidence-based guideline consensus, hypertension with elevated homocysteine (Hcy, Hcy ≥ 10 umol/L) is defined as hyperhomocysteinemia (H-type) hypertension [82]. High serum-Hcy and low plasma-folate are common in Chinese hypertensive patients [83]. In addition, the 677TT genotype of the methylene tetrahydrofolate reductase (*MTHFR*) gene, a key enzyme in the folate metabolic pathway, is frequently found in the Chinese population, resulting in an elevated Hcy level [84]. High serum-Hcy is an independent risk factor for vascular disease, which is closely associated with the development of hypertension [85,86]. Moreover, a meta-analysis has shown that Hcy was significantly associated with the risk of stroke and coronary heart disease [87]. Mechanistically, elevated homocysteinemia diminishes vasodilation by nitric oxide, increases oxidative stress, impairs vascular-endothelial function, and alters the elastic properties of the vessel wall. These factors collectively contribute to elevated BP [88,89]. In 2017, a meta-analysis including 11 studies showed a positive association between serum-Hcy levels and the risk of hypertension [90]. Similarly, a cross-sectional study demonstrated that every 5 umol/L increase in Hcy was associated with a systolic/diastolic BP increase of 0.45/0.47 mmHg in Chinese adults [91]. However, the above study does not support a causal relationship between Hcy levels and hypertension. Further prospective studies need to be conducted.

### 7.2. Diagnosis and Hypertension-Mediated Organ Damage

All hypertensive patients should be tested for serum-Hcy level. In addition, plasma-folate levels and the *MTHFR* 677TT genotype should also be examined. In terms of hypertension-mediated organ damage, a high serum-Hcy level was related to arterial stiffening and renal dysfunction in elderly Chinese [92]. A retrospective study in China also found that urinary microalbumin/creatinine and carotid-artery thickness were elevated in patients in H-type hypertension group compared to the normal hypertension group [93]. Moreover, Sun et al. found that high serum-Hcy was significantly associated with all-cause mortality and cardiovascular events in a prospective cohort study [94].

### 7.3. Treatment

Patients with H-type hypertension need to consume more folic-acid-rich foods in addition to general-lifestyle interventions and anti-hypertensive drugs. A meta-analysis in 2016 revealed high-risk factors for H-type hypertension and highlighted the importance of folic acid, B vitamins, fruits and vegetable supplementation, regular exercise, and smoking cessation [95]. Yong Huo et al. found that the combined use of enalapril and folic acid reduced the risk of a first stroke by 21%, compared to enalapril alone in the Chinese population with hypertension [96]. The American Heart Association/American Stroke Association also recommended the use of folic acid and vitamin B for stroke prevention in patients with high levels of serum Hcy [97].

## 8. Conclusions and Prospects

With the trend in global aging, the prevalence of hypertension is increasing annually, so it is particularly important to have an early diagnosis and proper treatment of hypertension. In the era of precision medicine, in addition to the development of a standardized approach to the treatment of hypertension based on the current clinical evidence, it is more effective to perform a personalized assessment of the primary mechanism of each hypertensive patient and then to select the preferred medicine, accordingly. However, the application of overly complex tests is not practical or suitable for routine practice. Therefore, based on the current evidence, we summarized the six categories of hypertension, to facilitate hypertension management. Nevertheless, there are also many limitations in this classification. On the one hand, there is a lack of sufficient clinical studies to confirm its validity, and on the other hand, some studies have concluded that certain classifications are not suitable for clinical application. However, it is hoped that the theoretical classification will further promote high-quality studies on different mechanisms of hypertension, which may contribute to more selective treatment. 

## Figures and Tables

**Table 1:** Recommendations for the etiological typing of secondary hypertension and its examination and treatment.

Etiological Subtype	Clinical Clues	Screening Method	Further Diagnostic Tests	Treatment
Nephrogenous hypertension	Personal/family history of chronic kidney disease; frequent urination, nocturia.	Urine routine; serum creatinine.	renal ultrasonography	Drug therapy: ACEIs or ARBs (first-line medication); ACEIs or ARBs + dihydropyridine CCB, ACEIs or ARBs + thiazide diuretics and dihydropyridine CCB + thiazide diuretics (combined therapy).
Renovascular hypertension	Murmurs in the abdomen or in other arteries; moderate-to-severe hypertension (grade 2 or above) and unilateral small kidney/transient pulmonary edema; young (<30 years) hypertensive patients with fibromuscular dysplasia; atherosclerosis.	Serum creatinine; renal artery ultrasound.	CT angiography; gadolinium contrast agent + MRA; renal angiography	Drug therapy: antihypertensive drugs, antiplatelet drugs and lipid-lowering drugs; non-drug treatment: percutaneous stent implantation, balloon dilatation.
Primary aldosteronism	Symptoms of hypokalemia;refractory hypertension;adrenal adenoma;family history of primary aldosteronism and/or early-onset hypertension or cerebrovascular accident (< 40 years old.)	Serum potassium; Aldosterone/renin ratio (ARR).	Saline load test; captopril test; adrenal CT scan; blood was collected from bilateral adrenal veins	Drug therapy: spironolactone is the first choice for bilateral lesions and eplerenone can be used for intolerance; non-drug treatment: surgical resection is the first choice for unilateral lesions.
Pheochromocytoma	Headache, palpitations, sweating, fainting; unstable blood pressure; paroxysmal hypertension.	Blood and urine catecholamine levels; blood and urine 3-Methoxyadrenaline.	CT/MRI; nuclear medicine imaging genetic testing	First-line treatment: surgical resection; second-line treatment: radionuclide therapy, radiotherapy or chemotherapy.
Cushing’s syndrome	History of osteoporosis at a young age; central obesity, purple skin lines, full-moon face, buffalo back.	24 h urine-free cortisol measurement; midnight salivary-cortisol measurement; adrenocorticotropic hormone.	Low-dose dexamethasone suppression test; large dose dexamethasone suppression test; CRH stimulation test; CT or MRI	Surgical resection; radiotherapy or chemotherapy; adjuvant therapy with drugs.
Aortic stenosis	The systolic blood pressure of upper limb is 20 mmHg more than lower limb; headache; delay or disappearance of femoral beats; arterial murmur.	Echocardiography	CTA or MRA	Endovascular aortic intervention.
Sleepapnea syndrome	Snoring; daytime sleepiness; asthma, asphyxia or apnea during sleep at night.	Sleepiness scale; history of snoring	Polysomnography	First-line treatment: non-invasive positive airway pressure ventilation; second-line treatment: oral orthotics, surgical treatment; etiology and lifestyle intervention: weight loss, smoking cessation, etc.

ACEIs: angiotensin-converting enzyme inhibitors; ARBs: angiotensin-converting enzyme inhibitors; CCB: calcium channel blocker; CT: computed tomography; CTA: computed tomography angiography; CRH: corticotropin-releasing hormone; MRI: magnetic resonance imaging; MRA: magnetic resonance angiography.

## Data Availability

Not applicable.
